# Invasive Plants and Species Richness Impact Litter Decomposition in Riparian Zones

**DOI:** 10.3389/fpls.2022.955656

**Published:** 2022-07-07

**Authors:** Xin Hu, Muhammad Arif, Dongdong Ding, Jiajia Li, Xinrui He, Changxiao Li

**Affiliations:** ^1^Key Laboratory of Eco-Environments in the Three Gorges Reservoir Region (Ministry of Education), Chongqing Key Laboratory of Plant Resource Conservation and Germplasm Innovation, College of Life Sciences, Southwest University, Chongqing, China; ^2^Biological Science Research Center, Academy for Advanced Interdisciplinary Studies, Southwest University, Chongqing, China

**Keywords:** Three Gorges Reservoir, hydro-fluctuation zone, exotic plants, species diversity, submergence environment, plant traits

## Abstract

Natural ecosystems generally include litter decomposition as part of the natural cycle since the material properties and the environment greatly influence the decomposition rate. The invasion of exotic plants alters the species diversity and growth characteristics of plant communities, but its impact on litter decomposition is unknown in the riparian zone. This study examines how invasive plants affect the early stages of litter decomposition and how species richness impacts them. This experiment involved a random litter mixture of exotic (*Alternanthera philoxeroides* and *Bidens pilosa*) and native species in the riparian zone of the Three Gorges Dam Reservoir in China. There were 43 species mixture types, with various species richness ranging from 1 to 6. Litterbags were placed in the hydro-fluctuation zone and terrestrial zone, where they decomposed over the course of 55 days. Invasive plants decompose rapidly compared to native plants (35.71% of the remaining mass of the invasive plant). The invasive plant *A. philoxeroides* has the potential to accelerate native plant decomposition (0.29 of non-added synergetic effect), but *Bidens pilosa* cannot. Nonetheless, species richness had little effect on the decomposition rate. These effects are dependent upon differences in chemical functional characteristics among the species. The initial traits of the plants, specifically C, N, and C/N, were significantly and linearly correlated with the loss of mixed litter mass and mixing effect strength (*P* < 0.01). In addition, submergence decomposition conditions reduce the disturbance of invasive plants and predict decomposition rates based on litter characteristics. Invasive plants can therefore impact the material cycle of an ecosystem. There is a need to examine decomposition time, which may also involve considering other factors.

## Introduction

Litter decomposition plays a crucial role in determining ecosystem function (Chen et al., 2021; Zhang et al., 2021). It is an important link in the material cycle and nutrient flow that can continuously provide resources to the soil and plants (Scheffer and Aerts, 2000; Zhang et al., 2020). There is a rapid mass loss rate and nutrient release that increases the resource-use efficiency of the environment in the early decomposition stage (Zhou et al., 2021). The litter decomposition process is generally regulated by climatic patterns, soil nutrients, decomposer assemblages, and litter properties (Yelenik et al., 2007). Litter decomposition is considered the more important control factor. It has been shown that invasive plants decompose more rapidly due to their nitrogen-rich nature (Gao et al., 2016; Yang et al., 2022). While climate and environmental change caused the exotic species to invade, the variances in growth traits between exotic and native plants led to the invasive plant's entry into the plant community, altering the mean plant community traits, which may impact the litter decomposition rates (Ashton et al., 2005; Leffler et al., 2014). Invading plants typically have higher N content than native plants, and invertebrates and microorganisms favor a lower C/N proportion than native plants and the characteristic. Because of this, the plant litter from invasive plants is likely to break down more quickly. This quickly releases nitrogen and other nutrients into the soil, which changes the environment for the plants that have been taken over (Poulette and Arthur, 2012).

Due to the long-term coexistence of exotic plants and native plants, exotic plants will inevitably enter the native plant's community (Hamid et al., 2020). The effect of changing the plant community means accelerating or slowing down the decomposition rate of the litter mixture (Zhang et al., 2014). Many studies refer to the acceleration or slowing down of the litter mixture decomposition rate as a non-additive effect (Dai et al., 2020). The introduction of exotic plants into the native plant community has accelerated the mass loss and release of nitrogen from native plant litter (Liu et al., 2020). According to the nutrient transfer theory, material components and nutrient elements are transferred between different species during the litter mixture decomposition process. Due to the action of invertebrates and the formation of mycelium between the litter by fungal microorganisms, nutrients are transferred from high-quality to low-quality litter, thus increasing the nutrient content of low-quality litter and attracting more decomposing microorganisms to settle (Lopes et al., 2011; Purahong et al., 2016; Bani et al., 2018). Also, because invasive plants release secondary metabolites like polyphenols, the rate of litter decomposition can be slowed even if invasive plants break down faster than native plants (Hättenschwiler and Vitousek, 2000).

It is clear that invasive species change the average characteristics of a plant community, and scientists have found that invasive species fill niches in a plant community, which changes the number of species (Dawson et al., 2015). Species richness determines the structure and function of ecosystems, and it may predict the ecosystem material cycles (Wardle et al., 1997; Behzad et al., 2022). As species richness increases, the functional diversity of litter becomes complex, the decomposition microenvironment changes (Hector et al., 2000; Gul et al., 2022), and the decomposer community species diversity may subsequently change to affect decomposition rates (Lecerf et al., 2007). It has been shown that the reduction in plant litter and decomposer diversity slows down carbon and nitrogen cycling, and the main causes are litter quality and environmental (Wu et al., 2013; Handa et al., 2014). Therefore, we expected that the addition of invasive plants to the plant community, which have different traits than native plants, would impact litter decomposition.

Current studies on the relationship between exotic plant invasion and species diversity focus more on primary productivity and growth traits. A strong negative correlation between plant diversity and invasive plant performance indicates that high species diversity can resist exotic plant invasion (Naeem et al., 2000). As increased native species diversity alters the competitive environment in plants, it reduces the effect of light, decreases the effect of nutrients, and inhibits the growth of exotic species. However, when plants decay into litter after they die off, invasive species can rapidly decompose and release nitrogen nutrients, increasing soil resource use efficiency and causing more species invasion, achieving positive feedback regulation (Prescott and Zukswert, 2016). Moreover, mixing exotic plants with native plants accelerates the native plant's decomposition and nutrient release rate (Chen et al., 2013; Schuster and Dukes, 2014). Thus, the interconnection between native and invasive plants is more complicated. Most studies were based on exotic and native species mixtures to observe the results of mixed litter decomposition. There were few responses on the effect of the invasive plants on the native plant's litter decomposition in terms of biodiversity. Therefore, this research aimed to determine the effect of invasive species on litter decomposition at different species richness.

Ecosystems on land and in water are connected by the riparian zone. Moreover, it connects habitats with diverse forms of life and provides important ecosystem services (Ding et al., 2021; Zheng et al., 2021a; Arif et al., 2022a). Three Gorges Dam Reservoir (TGDR) is in a fragile ecological ecotone due to the hydro-fluctuation, leading to exotic plant invasion, which may affect the native plant's decomposition (Xiao et al., 2017; Li et al., 2021). In the TGDR, *Alternanthera philoxeroides* and *Bidens pilosa* are the main invasive species, and their invasion changes the species composition and community structure (Lee et al., 2014). Due to differences in species growth traits, there may be deviations in the response of the abundant native plants to invasive plants in the TGDR zone. The role of invasive plants may deviate from that of different species' richness. The following scientific hypotheses are presented in this study: (i) invading plants can accelerate litter decomposition in the TGDR zone under submergence conditions; (ii) species diversity can mitigate the impact of invasive plants on litter decomposition; and (iii) there can be a relationship between litter traits and decomposition rates. We conducted this study to determine how invasive plants affect litter decomposition and how long it takes for mixed litter to decompose.

## Materials and Methods

### Experimental Site

The experimental site was located in the Ruxi River basin, Shibao Town, Zhongxian County, Chongqing City, TGDR (30°36′ N, 108°06′ E). The TGDR is the largest reservoir in China, located between Jiangjin in Chongqing and Yichang in Hubei, with an area of 349 km^2^. The TGDR implements a hydrological dispatching model of winter storage and summer discharge, creating a hydro-fluctuation zone with a water level difference of up to 30 m during 1 year (Sang et al., 2019; Arif et al., 2022b). The site is part of the subtropical southeast monsoon region's mountainous climate, with an average annual accumulated temperature of 18.2°C, long sunshine hours, sufficient rainfall, 1,327.5 h of sunshine, 29% sunshine rate, rainfall mostly concentrated between June and August, with an annual rainfall of 1,200 mm and relative humidity of 80 %. Herbaceous plants dominate the reservoir area. This area's soil is designated as purple soil (Regosols in FAO Taxonomy or Entisols in USDA Taxonomy) (Chen et al., 2020; He et al., 2021; Hu et al., 2021).

### Experimental Species

There are abundant herbaceous plants in the TGDR. We selected 6 native and 2 invasive plants with high dominance, high cover, and wide distribution as experimental species based on previous studies on the vegetation distribution in the TGDR (Table 1). These species include both annual and perennial herbaceous plants with a predominance of graminoid herbs, which is consistent with the vegetation in the water-level-fluctuation zone of TGDR. The two invasive plants were chosen to analyze the importance of species traits because variations in species traits may have different effects on litter decomposition rates.

**Table 1:** An overview of selected species used in the riparian zone of the Three Gorges Reservoir, China.

**Species**	**Family**	**Common name**	**References**
*Cynodon dactylon*	Graminaceae	Bermuda grass	Arif et al., 2021; Yuan et al., 2021; Zheng et al., 2021a
*Hemarthria altissima*	Graminaceae	Bullwhip grass	Zhong et al., 2016; Xiong et al., 2018; Liu et al., 2019
*Xanthium sibiricum*	Asteraceae	Siberian cocklebur	Sun et al., 2011; Mi et al., 2016; Liu et al., 2019
*Physalis alkekengi*	Solanaceae	Winter cherry	Zhu et al., 2015; Zhang et al., 2016; Chen et al., 2022
*Polygonum lapathifolium*	Polygonaceae	Polygonum scabrum Moench	Zhu et al., 2015; Deng et al., 2017; Zheng et al., 2021b
*Digitaria sanguinalis*	Graminaceae	Crab grass	Zhu et al., 2015; Zheng et al., 2021a; Chen et al., 2022
Alternanthera philoxeroides^*^	Amaranthaceae	Alligator weed	Zhu et al., 2015; Zhang et al., 2016; Deng et al., 2017
Bidens pilosa^*^	Asteraceae	Sticktight	Zhu et al., 2015; Mi et al., 2016; Chen et al., 2022

* *Sign used for the invasive plant in the riparian zone of the Three Gorges Reservoir, China*.

### Experimental Design

The above-ground parts (stems and leaves) of eight herbaceous plants were collected from experimental site area in 2021. Experimental material was frozen and quickly brought back to the laboratory, chopped into 10 cm-sized pieces, and dried to a constant weight of 80°C.

Litterbags contained one of 43 mixture types (or combinations). This litter mixture type includes four species numbers. Species richness 1 is 8 monocultures, and 2, 4, and 6 species mixture litterbags include 3 classifications: (1) Combination of native and native species: native-group. We randomly selected five 2-species mixtures from all possible 2-native species combinations in the 6-native species pool. The selection method for the 4-species mixture is the same as above. The 6-species mixture was not selected because it includes all species. (2) Combination of native and invasive species *A. philoxeroides*: Ap-group. Six 2-species mixtures of Ap-group are all possible combinations of the 6-native species and the *A. philoxeroides* pool. To study the impact of invasive plants on litter mixture and the possibility of comparing native-group and AP-group without changing species richness, we substituted *A. philoxeroides* randomly from one native species of the selected native-group. (3) Combination of native and invasive species *B. pilosa*: Bp-group. To study the possible effects of different species litter mixtures, although they are both invasive plants, 2, 4, and 6-species mixtures of the Bp-group, we replaced *B. pilosa* with *A. philoxeroides* of the Ap-group. The number of species combinations is shown in Table 2. According to the combination design, the sample dried to constant weight was packed evenly weighted into nylon decomposition bags (15 × 20 cm, aperture 1 × 1 mm). Each decomposition bag was filled with a total of 6.0 g of sample (e. g., 2-species mixture litterbags contained 3.0 g of litter from each species and 4-species mixture litterbags contained 1.5 g of litter from each species). We set up 6 replicates of each mixture type, and 258 litterbags were prepared.

**Table 2:** The number of species combinations used in the riparian zone of the Three Gorges Reservoir in China.

**Species richness**	**Native group**	***A. philoxeroides* group**	***B. pilosa* group**	**Total**
1	6	1	1	8
2	5	6	6	17
4	5	5	5	15
6	1	1	1	3

*Source: Lecerf et al. (2007), Hickman et al. (2013), and Grossman et al. (2020)*.

We selected an experimental transect in the water-level-fluctuation zone of the TGDR, which is perpendicular to the river and has rich vegetation on the ground. We selected two sample points in the hydro-fluctuation zone (S) and terrestrial zone (CK) as experimental points in the transect. The highest level of water in the TGDR was 175 m. Our experimental points in the S and CK were, respectively, set at 170–175 m and 175–180 m. Although the water level fluctuated slightly during the experiment, it always flooded the litter bags at the experimental points. We cleared each experimental point's surface vegetation and debris and formed a 2 × 3 m experimental plot. Before the water level rose, the litterbags were carefully placed at the two experimental points to ensure that the arrangement between the decomposition bags was close and not overlapping. Start the experiment when the water level has raised and soaked the litterbags at S. The litterbags at S were flooded for a period of time. When the water level declined and the litterbags were exposed, all the litterbags at the two experimental plots were brought back to the laboratory (each litterbag was packed independently in a valve bag).

### Sample Processing and Measurement Methods

The decomposition experiment started in October 2021, and the decomposition bags were retrieved after the water level dropped, decomposing for 55 days. After bringing the decomposition bags back to the laboratory, the surface contaminants were carefully removed. The impurities were rinsed three times with ultrapure water after being progressively rinsed with tap water. The washed litter was placed in weighted and marked envelopes, dried to a constant weight in an oven at 80°C, and then weighed. The initial samples were pulverized in a ball mill, passed through a 0.25 mm sieve, and used for chemical composition determination.

The initial C and N contents were determined using an elemental analyzer (CHNS-O-Vario EL Cude; Heraeus Elementar, Hanau, Germany), and Ca, Mg, and P were first digested by microwave digestion (SpeedWave MWS-4) and then determined by inductively coupled plasma emission spectrometry (ICP-OES, Thermo Fisher Scientific iCAP 6300).

### Data Processing and Analysis

The formula for calculating the remaining rate based on mass is as follows (Voříšková and Baldrian, 2013):


$$\begin{array}{l} \text{Remaining Mass rate}={\text{M}}_{\text{T}}/{\text{M}}_{\text{O}}\times 100\text{\%} \end{array}$$

Where M_0_ denotes the sample's initial mass; M_T_ denotes the sample's weight after the time T.

The mixing effect intensity of litter decomposition is estimated by comparing the expected value with the actual value of litter mass loss. According to the mixing effect intensity, we can infer what kind of mixing effect (additive effect, synergistic effect, or antagonistic effect) exists in the litter mixture decomposition process. The expected decomposition rate of mixed litter is calculated from the actual decomposition rate of individual species under the summation effect and is calculated as follows (Lecerf et al., 2007):


$$\begin{array}{l} E=\frac{\sum_{i=1}^{n}\left(DM_{i}\times O_{i}\right)}{\sum_{i=1}^{n}DM_{i}} \end{array}$$

where *E* is the remaining mass expectation value for litter decomposition for different litter mixtures. *DM*_*i*_ is the initial mass of species *i*; *O*_*i*_ is the measured remaining mass rate of species *i* alone.

The mixing effect intensity of litter decomposition was calculated from the actual observed and expected values of litter decomposition rate by the following equation (Chen et al., 2013):


$$\begin{array}{l} \text{Mixing effect intensity}=\\ 1-\left(\text{observed value}/\text{expected value of remaining mass}\right) \end{array}$$

For each of these chemical features, we estimated functional trait identity and diversity for all litter mixture elements. The abundance-weighted mean of the trait value across all constituent species in a community, known as the community-weighted mean (CWM), is used to demonstrate community-level trait identity for a given trait and community. Because all species were represented in equal abundance, CWM equaled the mean in this situation. For all features across all communities, we estimated functional dispersion, a marker of functional diversity. The functional dispersion for one trait is highest when species are maximally dissimilar for this trait (Laliberte and Legendre, 2010). The following equation calculated the community weighted mean (CWM) and functional dispersion (FDis) of the litter mixture (Grossman et al., 2020):


$$\begin{array}{l} CWM=\overset{S}{\underset{i=1}{\sum }}Pi\times ti \end{array}$$

Where *Pi* is the relative richness of species *i, ti* is the trait value of species *i*, and *S* is the number of species in the community.


$$\begin{array}{l} C=\frac{\sum ai\cdot Xij}{\sum ai},\;FDis=\frac{\sum ai\cdot Zi}{\sum ai} \end{array}$$

Where *c* is the weighted centroid in the *j*-dimensional space, *ai* is the abundance of species *i, xij* is the attribute of species *i* for trait *j*, and *zi* is the distance of species *i* to the weighted centroid *c*.

The data was analyzed using SPSS 22 (IBM, Chicago, USA) and Microsoft Excel. ANOVA was used to analyze the effect of species richness, environment, and invasive plants on litter mass loss (α = 0.05). Paired *t*-tests were used to test the variation between expected and observed values for litter decomposition and monoculture remaining mass difference in two environments (α = 0.05). Linear correlation was used to investigate the link between the CWM and FDis of litter mixture with the mass loss and the mean mixing effect intensity. R was estimated for CWM and FDis. OriginPro 2022 (Northampton, MA, USA) was used for mapping.

## Results

### Impact of Invasive Species on Individual Native Species

**Figure 1:**
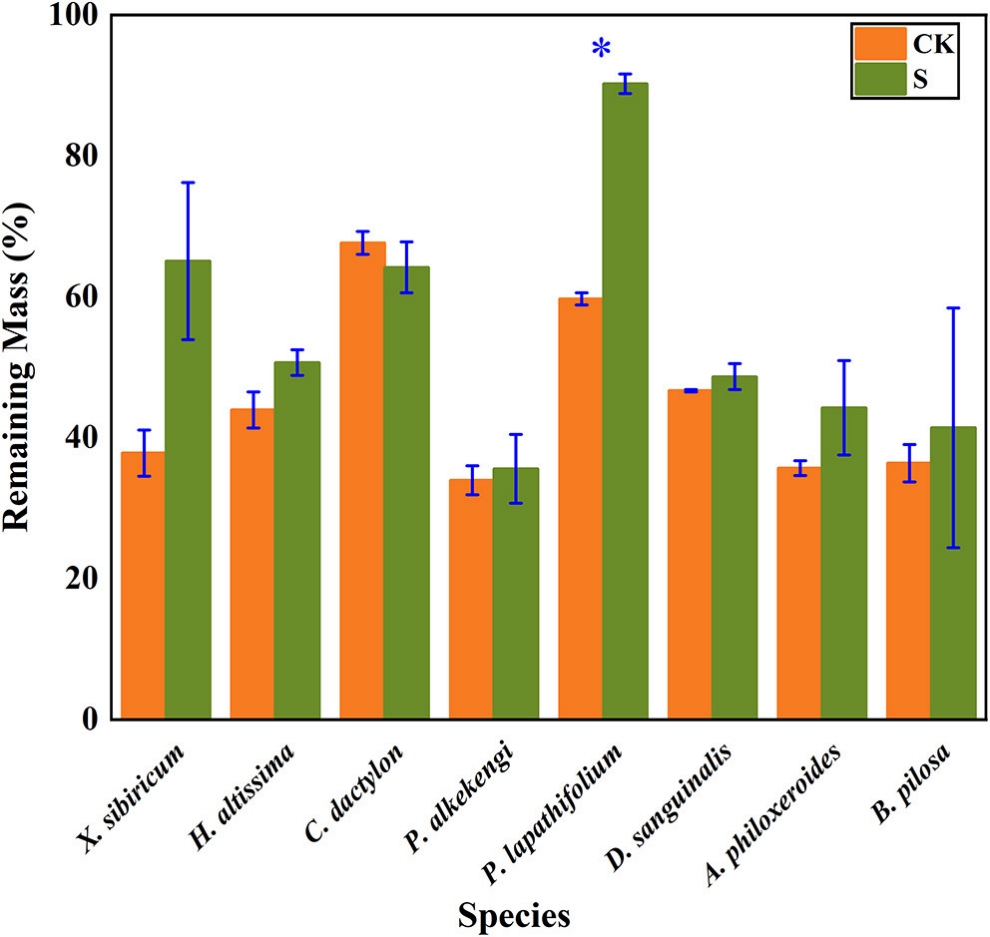
Bar charts show individual species' mass remaining litter rates within the hydro-fluctuation zone (S) and the terrestrial zone (CK). The *y-axis* shows the mean value (%). The *vertical whisker* represents the standard deviation. Paired tests were used to determine the differences between S and CK. ^*^Indicates significant differences at *P* < 0.05.

The litter's remaining mass rates were significantly affected by the species, submergence environment, and their interactions (*P* < 0.001). According to the individual species' litter remaining mass rate (Figure 1), the litter remaining mass at the CK and S were not significantly different, except for *P. lapathifolium*. *X. sibiricum, C. dactylon*, and *P. lapathifolium* decomposed more slowly, and the remaining mass rate at S was >60%, among which *P. lapathifolium* had the highest remaining mass. The remaining mass rate of *P. alkekengi, A. philoxeroides*, and *B. pilosa* was lower, at about 35% in both environments. According to the litter's initial elemental content (Table 3), the C content of all species was not very different, but the N element content of *A. philoxeroides*, and *B. pilosa* was high, up to 33.1 mg·g^−1^. *Alternanthera philoxeroides* had a lower C/N of 12.1 compared to other species. The N, P, Ca, and Mg element content of *P. alkekengi* was higher than that of the native species. The two-way ANOVA results for the litter remaining mass rates of the eight individual species are shown in Table 4.

**Table 3:** The initial elemental content of litter of selected species in the riparian zone of the Three Gorges Reservoir in China.

**Species**	**Indicators of chemical elements**					
	**Ca (mg·g^**−1**^)**	**Mg (mg·g^**−1**^)**	**P (mg·g^**−1**^)**	**N (mg·g^**−1**^)**	**C (mg·g^**−1**^)**	**C/N**
*C. dactylon*	19.44 ± 1.45	5.26 ± 0.28	10.76 ± 2.45	15.20 ± 3.81	392.67 ± 7.96	27.21 ± 8.26
*H. altissima*	2.50 ± 0.32	1.70 ± 0.11	4.69 ± 0.34	9.93 ± 1.11	424.0 ± 3.48	43.02 ± 4.48
*X. sibiricum*	3.66 ± 0.47	1.18 ± 0.09	4.27 ± 0.18	8.67 ± 0.55	411.70 ± 1.15	47.63 ± 3.07
*P. alkekengi*	21.18 ± 2.11	5.56 ± 0.03	17.07 ± 0.67	19.93 ± 0.58	385.40 ± 2.30	19.34 ± 0.46
*P. lapathifolium*	11.88 ± 0.69	2.36 ± 0.08	4.43 ± 0.20	13.43 ± 2.47	430.90 ± 2.48	32.87 ± 6.49
*D. sanguinalis*	4.45 ± 0.40	4.74 ± 0.65	5.24 ± 0.48	8.80 ± 0.35	389.37 ± 6.05	44.28 ± 1.15
*A. philoxeroides*	9.89 ± 0.58	3.17 ± 0.17	5.13 ± 0.26	33.10 ± 0.85	400.43 ± 1.85	12.10 ± 0.27
*B. pilosa*	15.71 ± 0.06	4.06 ± 0.03	8.77 ± 0.03	21.10 ± 0.52	425.83 ± 2.85	20.19 ± 0.37

*Mean ± standard deviation*.

**Table 4:** Two-way ANOVA results indicate the loss of litter mass of individual species in the riparian zone of the Three Gorges Reservoir in China.

**Source**	**Remaining litter mass**		
	**df**	***F***	***P***
Species	7	34.449	<0.001
Submergence	1	34.158	<0.001
Species × submergence	7	6.845	<0.001

**Figure 2:**
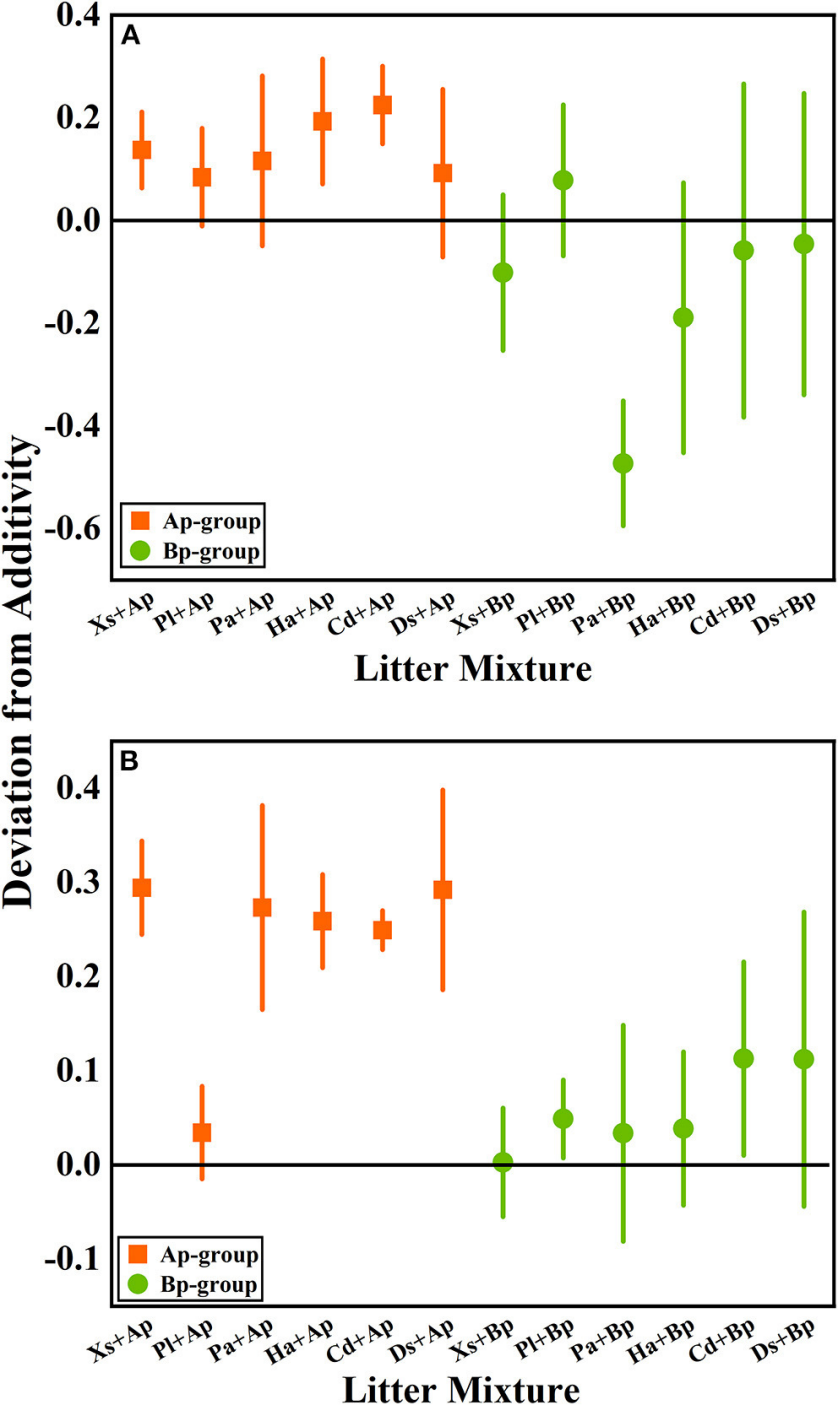
The mean mixing effect intensity of litter mixture mass remaining in the hydro-fluctuation zone **(A)** and terrestrial zone **(B)**. The Ap-group indicates litter mixtures of *A. philoxeroides* and native species, and the Bp-group represents litter mixtures of *B. pilosa* and native species. All data are presented as mean ± standard deviation. In the mean mixing effect intensity values, values above 0 indicate synergistic effects, and values below 0 indicate inhibitory effects. Xs, *X. sibiricum*; Pl, *P. lapathifolium*; Pa, *P. alkekengi*; Ha, *H. altissima*; Cd, *C. dactylon*; Ds, *D. sanguinalis*; Ap, *A. philoxeroides*; Bp, *B. pilosa*.

According to the mean mixing effect intensity of litter decomposition (Figure 2), it can be seen that the litter decomposition mixing effect in submergence and terrestrial environments is similar, but the impact degree is different. The Ap-group's mean mixing effect intensity value is >0, indicating that *A. philoxeroides* has a non-additive effect on the native plants' litter decomposition. But the effect intensity value in the terrestrial zone is higher than in submergence, indicating that the non-additive effect of *A. philoxeroides* under terrestrial conditions is greater. The mean effect intensity values for the Bp-group were around 0, indicating that *B. pilosa* does not have a non-additive effect on the native plants' litter decomposition.

### Effect of Invasive Species on Litter Decomposition on Different Species Richness

**Figure 3:**
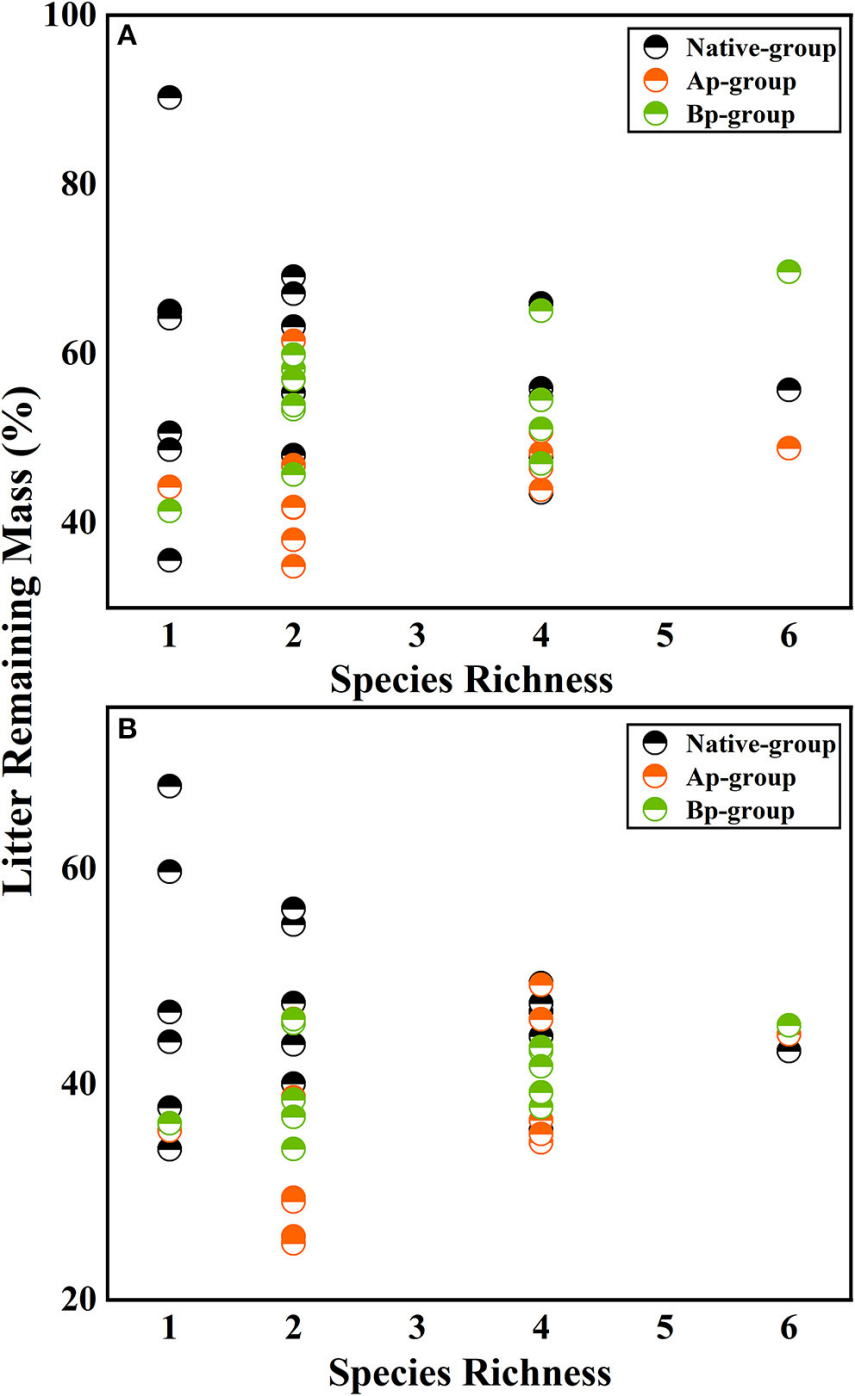
The litter remaining mass at different rates for different levels of species richness in the hydro-fluctuation zone **(A)** and terrestrial zone **(B)**. The Ap-group indicates litter mixtures of *A. philoxeroides* and native species, and the Bp-group represents litter mixtures of *B. pilosa* and native species. Data are expressed as mean values.

On the basis of the three-way ANOVA results (Table 5) and the litter remaining mass rate at different species richness levels (Figure 3), it can be concluded that the submergence environment and invasive species greatly contributed to litter decomposition (*P* < 0.001). The interaction between species richness and invasive plants significantly affected litter decomposition (*P* < 0.05). As species richness increased, the difference in remaining mass rate between every litter combination gradually decreased. The extreme difference in individual species' remaining mass rates reached 54.66% in the hydro-fluctuation zone. The extreme difference in mass remaining rate for species richness 2 decreased to 34.24%, followed by 22.32 and 20.84% for species richness 4 and 6, respectively. A similar trend result was shown in the terrestrial zone, with the extreme difference in mass remaining rate from 31.97% for the species richness 1, decreasing gradually to 2.39% for the species richness 6.

**Table 5:** Three-way ANOVA results for litter mass remaining rates on different species richness in the riparian zone of the Three Gorges Reservoir in China.

**Source**	**Remaining litter mass**		
	**df**	***F***	***P***
Species richness	3	1.264	0.287
Submergence	1	31.186	**<0.001**
Invasive species	1	15.463	**<0.001**
Species richness × submergence	3	1.159	0.326
Species richness × invasive species	3	3.099	**0.027**
Submergence × invasive species	1	0.124	0.725
Species richness × submergence × invasive species	3	0.269	0.848

*Bold values indicate significant differences between the results*.

**Figure 4:**
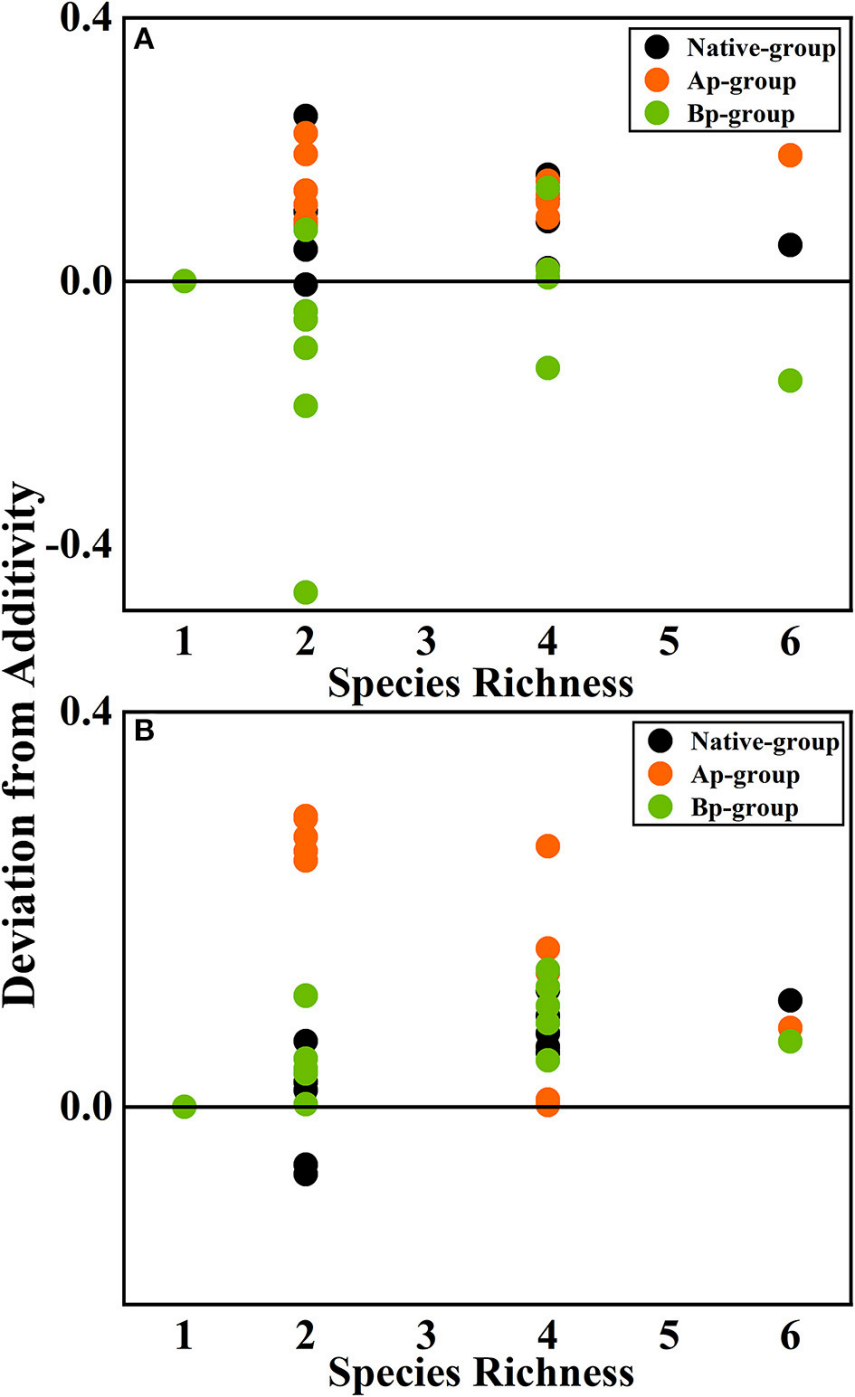
The mean mixing effect intensity of litter mixture remaining mass on different species richness in the hydro-fluctuation zone **(A)** and terrestrial zone **(B)**. The Ap-group indicates litter mixtures of *A. philoxeroides* and native species, and the Bp-group represents litter mixtures of *B. pilosa* and native species. Data are expressed as mean values. In the mean mixing effect intensity values, values above 0 indicate synergistic effects, and values below 0 indicate inhibitory effects.

**Figure 5:**
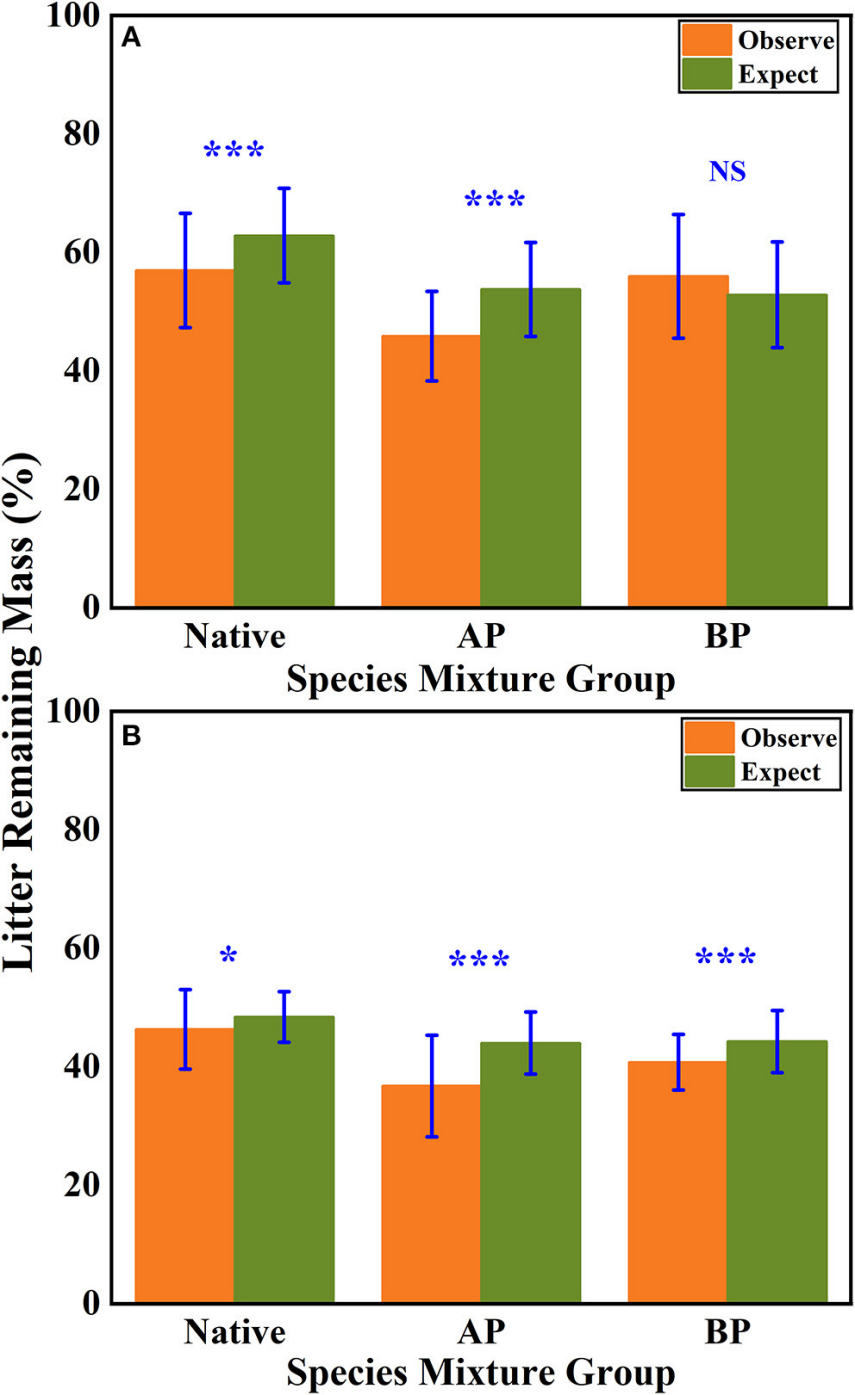
The results of the paired t-test show the litter's remaining mass rate at observed and expected values for the hydro-fluctuation zone **(A)** and terrestrial zone **(B)**. The Ap-group indicates litter mixtures of *A. philoxeroides* and native species, and the Bp-group represents litter mixtures of *B. pilosa* and native species. All data are presented as mean ± standard deviation. A paired *t*-test was used to test for significant differences between observed and expected values. NS, no significant difference; *P < 0.05; ***P < 0.001.

According to the mean mixing effect intensity for different litter species richness (Figure 4) and the paired *t*-test results of litter remaining mass rate observed and expected values (Figure 5), the mean mixing effect on different species richness has similar results, and the mixing effect intensity of the AP-group is higher than the 0 value. Still, the effect value gradually increases with the species richness. But the mixing effect intensity values of BP-group litter did not change significantly with the species richness. They were all around the 0 lines, indicating that the effect of the litter decomposition was relatively small. The decomposition environment difference significantly affected the mixing effect, with a significant difference (*P* < 0.001) between the observed and expected values for the Native-group litter on submergence conditions in the hydro-fluctuation zone but not for the combination mixed with *B. pilosa*. In contrast, the opposite result existed in the terrestrial zone. In addition, the AP-group litter observed values were significantly different from the expected values in both environments (*P* < 0.001), indicating that the effect of *A. philoxeroides* on the litter decomposition was highly significant and synergistic. Overall, the AP-group litter remaining mass rate was lower than the Native-group and BP-group.

### Correlation of the Litter Decomposition Rate and Mixing Effects Intensity With Litter Chemical Functional Traits

**Figure 6:**
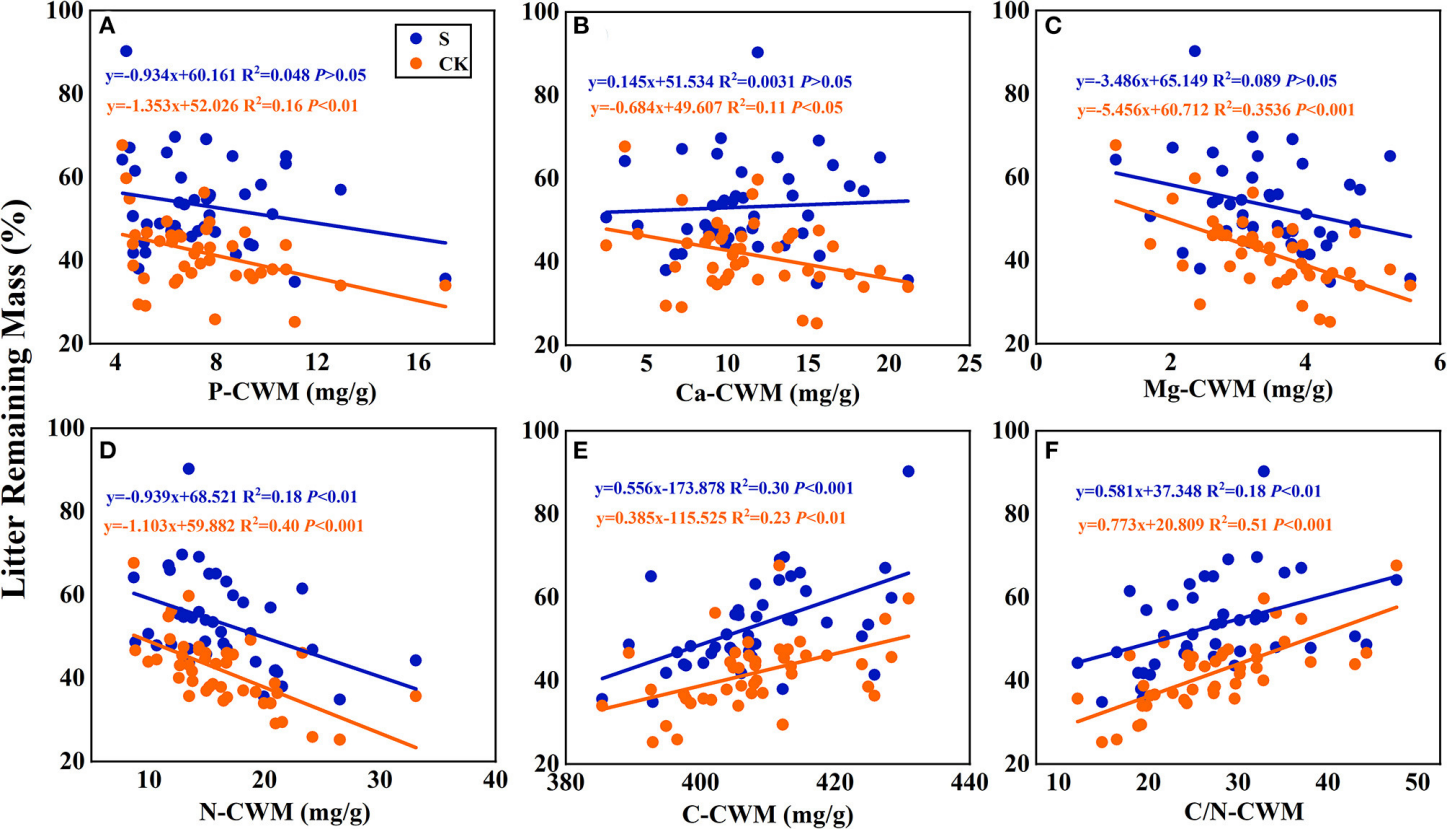
Linear regression relationships **(A–F)** between litter remaining mass rate and litter community weighted mean traits (CWM). S stands for litter that has been decomposed in the submergence environment, and CK stands for litter that has been decomposed in the terrestrial environment.

**Figure 7:**
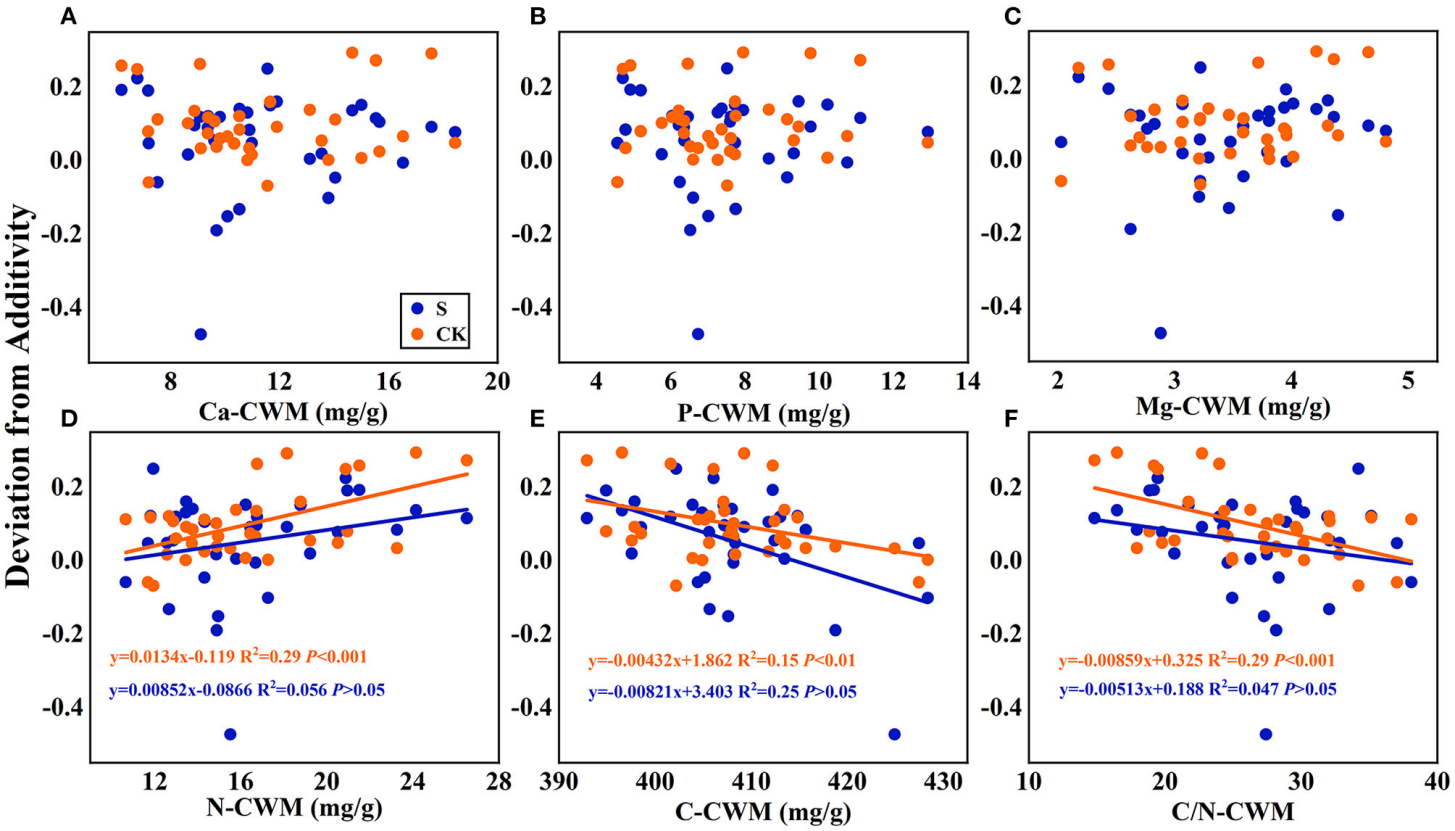
Linear regression relationships **(A–F)** between the mean mixing effect intensity of litter decomposition and community weighted mean traits (CWM) of litter mixture. S stands for litter that has been decomposed in the submergence environment, and CK stands for litter that has been decomposed in the terrestrial environment.

According to the linear regression relationship between the litter remaining mass rate (Figure 6) and the mixing effect intensity of litter decomposition (Figure 7) with litter CWM, the litter carbon-CWM and nutrient elements CWM have a significant linear relationship with the remaining mass rate at the litter decomposition early stage (*P* < 0.05). In the terrestrial environment, there was a significant negative correlation between the litter's P, Ca, Mg, and N-CWM and the remaining mass rate (*P* < 0.05). At the same time, C and C/N-CWM showed a significant positive relationship (*P* < 0.05). However, the relationship between litter P, Ca, Mg-CWM, and the remaining mass rate was not significant in the submergence environment (*P* > 0.05), but C, N, and C/N remained significant.

Regarding the mean mixing effect intensity of litter decomposition, there was a highly significant linear correlation between C, N, and C/N-CWM and the mixing effect intensity in the terrestrial environment. The N-CWM showed a positive correlation with the mixing effect intensity (Figure 7D, y = 0.0134–0.119, *P* < 0.001), and C and C/N-CWM showed a negative correlation with the mixing effect intensity (Figure 7E, y = −0.00432x + 1.862, *P* < 0.01; Figure 7F, y = −0.00859x + 0.325, *P* < 0.001). However, there was no such significant linear correlation in the submergence environment (*P* > 0.05). In addition, there was no significant linear regression between Ca, P, and Mg-CWM and the mixing effect intensity in both environments.

**Figure 8:**
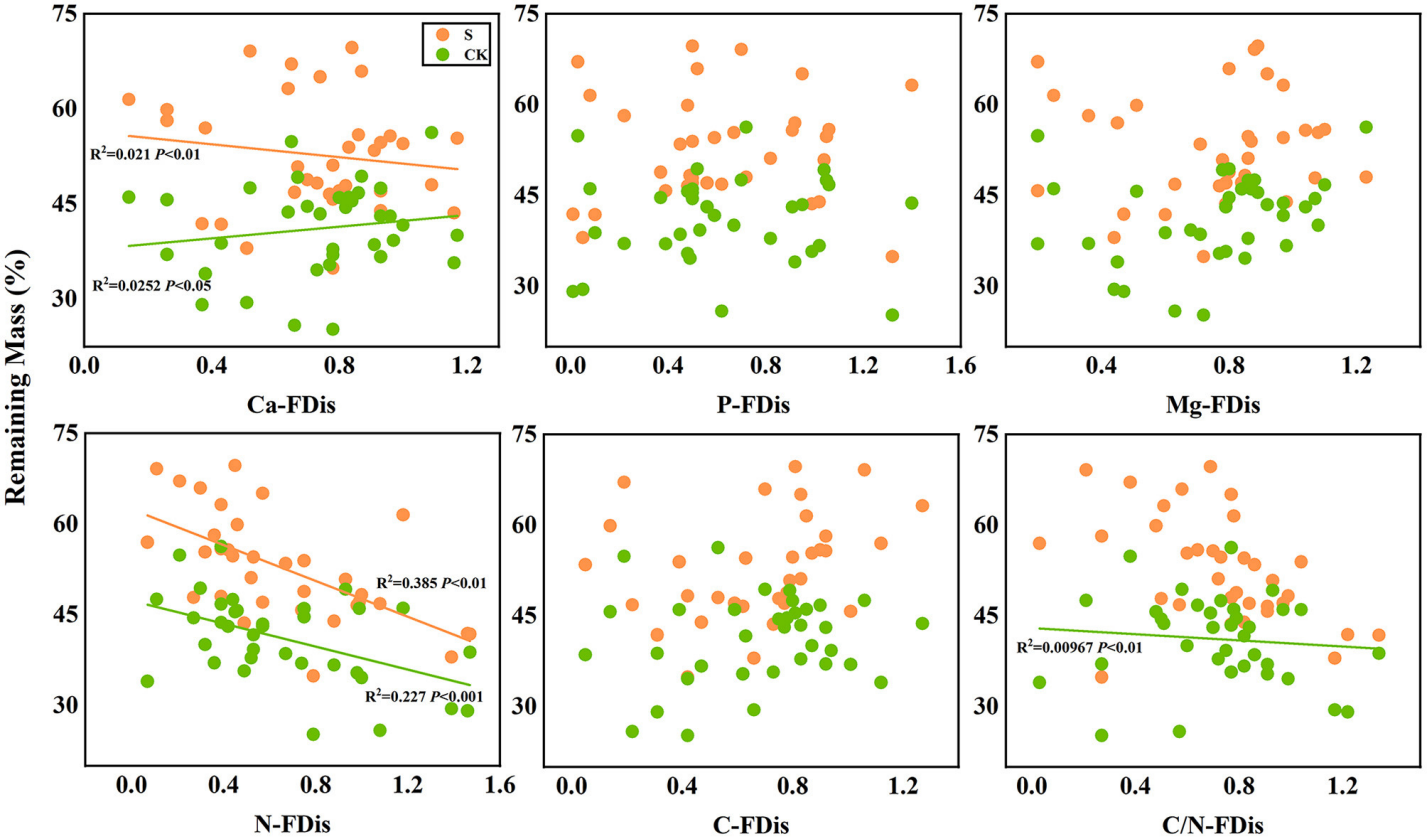
Linear regression relationships between litter remaining mass rate and litter functional dispersion (FDis). S stands for litter that has been decomposed in the submergence environment, and CK stands for litter that has been decomposed in the terrestrial environment.

**Figure 9:**
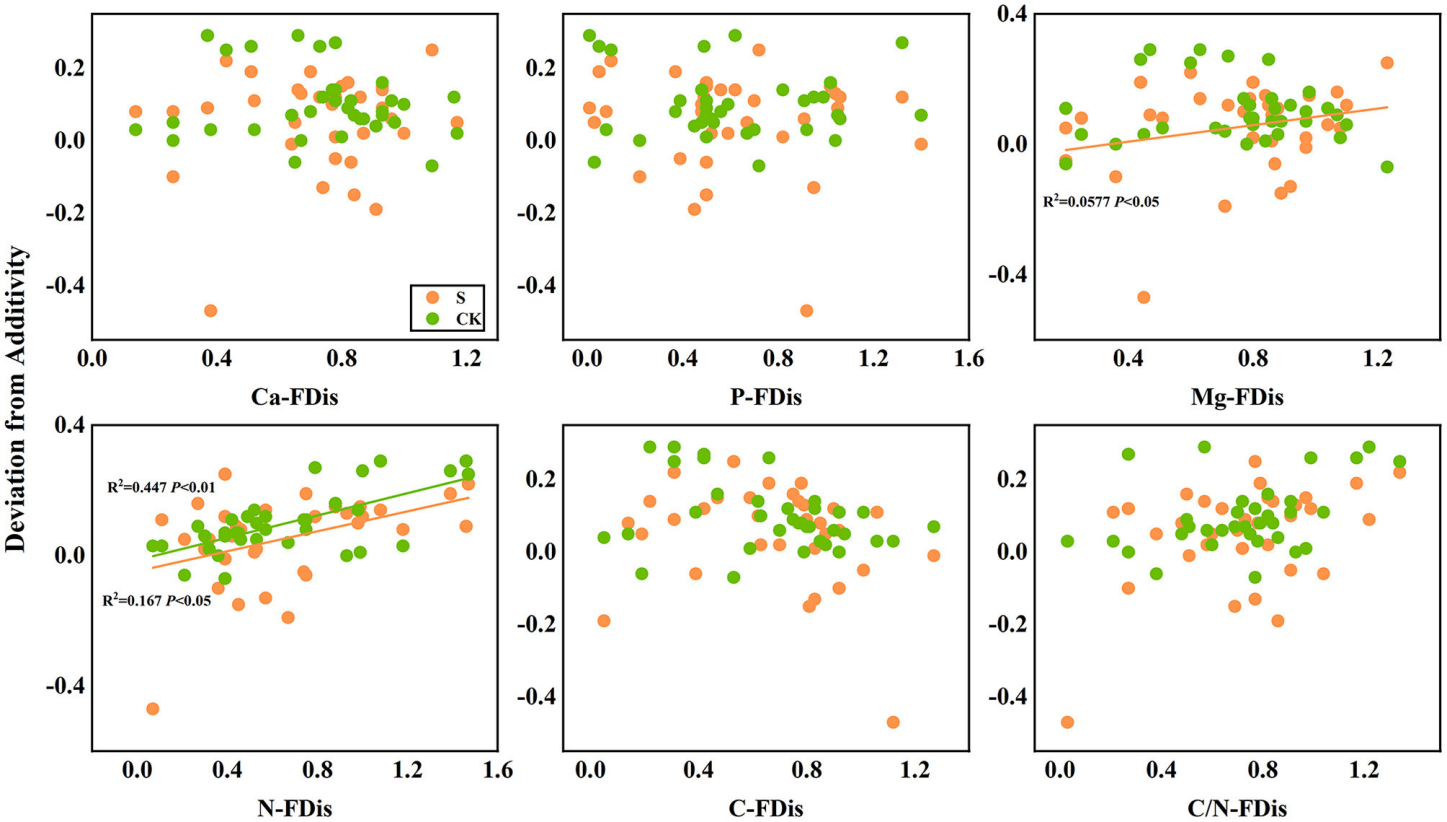
Linear regression relationships between mean mixing effect intensity of litter decomposition andfunctional dispersion (FDis) of litter mixture. S stands for litter that has been decomposed in the submergence environment, and CK stands for litter that has been decomposed in the terrestrial environment.

In addition, according to linear regression relationships between litter remaining mass rate (Figure 8) and the mean mixing effect intensity (Figure 9), and litter functional dispersion (FDis), N-FDis was significantly correlated with litter mass loss and litter mixing effect intensity (*P* < 0.05). This situation exists in both environmental conditions. Ca-FDis was significantly correlated with mass loss (*P* < 0.05), and C/N-FDis was extremely significantly correlated with decomposition in the terrestrial zone (*P* < 0.01).

## Discussion

Litter decomposition simplifies and slowly releases complex compounds into chemical elements, regulated by the litter quality and environmental conditions (Gessner et al., 1999). Typically, invasive species in a plant community have different chemical functions and traits from native species and may interfere with litter decomposition (Table 3). In this experiment, *A. philoxeroides* and *B. pilosa* are both invasive species, but they have different effects on native plant decomposition due to their respective chemical traits. The mixture of *A. philoxeroides* with native species accelerated mixed litter mass loss, showing a synergistic decomposition effect in submergence and terrestrial environments, which is consistent with hypothesis 1. However, there was no significant difference in litter mass loss from the mixture of *B. pilosa* and native species compared with the native monoculture, which showed an additive effect and a smaller effect on the native species' decomposition (Figure 2). The reason for *A. philoxeroides*'s accelerated litter decomposition may be the high N elemental content. Studies have shown that the rapid mass loss of invasive plants compared to native plants is related to the N element (Figure 1) (Gartner and Cardon, 2004; Incerti et al., 2018). Invertebrates and microorganisms feed on nutrients to meet growth needs, and decomposers prefer to feed on litter with high N content because N-containing components are a high-quality nutrient, resulting in a faster mass loss rate (Gessner et al., 2010). According to nutrient transfer theory, N and other nutrient elements can be transferred in litter, moving from high-quality to low-quality litter, thus accelerating the decomposition of low-quality litter and overall showing mixed litter rapid decomposition (Maisto et al., 2011). Enríquez showed a general positive linear relationship between litter N and P content and the decomposition rate during litter decomposition due to the high demand for these essential nutrients by decomposing organisms, which rapidly feed on the high-quality litter to meet their needs (Enríquez et al., 1993). Usually, the litter produced by different plant species differs in quality and attractiveness to decomposers, resulting in large differences in the mass and nutrient losses in different species and environments (Grossman et al., 2020).

In the early litter decomposition stage, a faster mass loss and nutrient release rate were strongly correlated with the nutrient content of the plant tissues (Debusk and Reddy, 2005; Zhang et al., 2019). The plant chemical functional traits related to the mixed litter decomposition are consistent with hypothesis 3, but this depends on the differences in material composition between litter species and the environment (Figures 6–9) (Hoorens et al., 2003). The growth traits and material composition of plants are the main characteristics that reflect the litter quality. Chemical elements exist in abundant forms of plant tissues, and the way of releasing chemical elements and the degree of attracting decomposing organisms are the litter decomposition control factors (Zhang et al., 2019). C is present in the plant in carbohydrates and tissues such as cellulose and lignin, which occupy most of the plant mass, so the litter C release rate is equal to the mass loss (Hu et al., 2021). Decomposing organisms prefer high-quality nutrient elements, and decomposing organisms prefer high-nitrogen substances to meet their growth (Wickings et al., 2012). Decomposing organisms have a higher demand for the N element. Therefore, litter with higher N content and lower C/N has a faster mass loss rate. And P is a turnover element in microbial growth and controls the normal growth of microorganisms (Webster and Benfield, 1986). Although the demand for trace elements is not as high as that for massive elements, they also play an indispensable role in regulating plant growth and metabolism, so discussing trace elements effectively predicts decomposition. Higher Ca content may accelerate litter mass loss because Ca is involved in some enzymatic activities and is more attractive to soil organisms (Grossman et al., 2020). Mg is an important component of chlorophyll and plays a key role in the fungi's growth (Cao et al., 2018).

Plant traits are highly correlated with litter mass loss in the mixed litter decomposition of invasive and native plants, but the increase in species richness is less discussed. As species richness increases, the decomposition process also becomes complicated. A complex mixed litter decomposition environment with more microbial species and diversity, higher litter heterotrophic respiration, and more invertebrate fauna may accelerate litter mass loss (Liu et al., 2007; Lecerf et al., 2011). However, the results suggested that species richness did not significantly affect litter decomposition (Smith and Bradford, 2003). However, the interaction between species richness and invasive species still had a significant effect (Table 5). Species richness had an effect in the invasive-native mixed litter but was not found in the native single litter. Several reasons may be for this (Liu et al., 2016): (1) Chemical traits do not differ significantly among native species. The main driver of mixture decomposition is still the average decomposability of component species (Figures 6, 8) (Harguindeguy et al., 2008). The chemical traits of native plants selected for this experiment did not differ significantly (mainly for N elements), resulting in essentially the likely initial traits of the native-native mixed litter at different species richness, showing no significant effect. Invasive plants significantly changed the mean chemical traits of mixed litter, so invasive plants significantly affected litter decomposition in every species richness. (2) This study focused on the rapid litter decomposition stage. Litter releases nutrients rapidly and is more susceptible to regulation by litter nutrient elements such as C/N. As litter enters a slow decomposition stage, the predictive role of nutrients decreases, the relevance of structural substances increases, such as lignin, and the effects of species richness may change (Grossman et al., 2020). (3) As decomposition proceeds, microbial diversity changes, which may be regulated by species richness (Voříšková and Baldrian, 2013; Kennedy and El-Sabaawi, 2017). Bardgett and Shine reported that moderate species richness (2–4) reduced microbial biomass, which may neutralize the effect of material heterogeneity, showing no significant effect on species richness overall (Bardgett and Shine, 1999).

Although the influence of species on decomposition is stronger, the environmental role cannot be ignored. Rapid litter decomposition is mainly due to microbial feeding and leaching, and different decomposition environments significantly affect the litter decomposition (Table 5) (Makkonen et al., 2012). The scouring of the river body first leaches out many soluble substances from the litter, resulting in rapid mass loss and nutrient release. The rapid nutrient release caused by water body scouring affected the predictive effect of the initial litter traits, resulting in a non-significant linear correlation between the litter initial P, Ca, and Mg elemental content and mass loss in the submergence environment (Figures 6–9). In addition, the aquatic environment involves more fungi. Some studies have shown that the contribution of fungal organisms in the litter decomposition process is greater than bacteria (Hieber and Gessner, 2002; Allison et al., 2013). The different nutrient requirements of the microbiota lead to variable attachment of microorganisms to litter, so the predictive role of nutrient elements may be even less in the aqueous environment (Chen et al., 2020). Waterbody nutrients also reduced the interspecific differences between plant litter and affected the predicted decomposition rate of mixed litter (Rosemond et al., 2010). Although both species and the environment impact litter decomposition, many studies have shown that the relative importance of species is stronger.

## Conclusion

There was a faster decomposition rate of invasive plants in the reservoir zone than native plants. There was a synergistic effect between *A. philoxeroides* and native plants on mixed litter decomposition, but the effect of *B. pilosa* was not significant. The litter decomposition rate did not differ significantly across species richness levels. However, the interaction between invasive plants and species richness greatly influenced litter mass loss. These results were associated with the initial chemical characteristics of the plants, and invasive plants altered the mean traits of plant communities. In particular, the carbon and nitrogen contents and the C/N ratio were linearly correlated with litter mass loss and mixing effect intensity. In addition, the submergence decomposition environment also mitigated the effect of invasive plants, particularly regarding water flushing and nutrients. In conclusion, invasive species accelerate the decomposition of native litter. However, this is influenced by the differences between the chemical functional traits of invasive and native plants. This experiment investigated the effects of overall plant trait differences and changes in species richness caused by invasive plants on litter mass loss during the rapid decomposition period. Furthermore, the decomposition time should be analyzed, which may involve several other factors in assessing the effects of invasive plants.

## Data Availability Statement

The raw data supporting the conclusions of this article will be made available by the authors, without undue reservation.

## Author Contributions

XHu, MA, and CL contributed to the conception and design of the study. XHu and MA organized the database. XHu, DD, JL, and XHe collected research samples and performed the statistical analysis. XHu, MA, and CL wrote and revised the manuscript. MA and CL supervised the research. All authors contributed to the article and approved the submitted version.

## Funding

This research has been supported by the Chongqing Municipality Key Forestry Research Project (No. 2021-9), Chongqing Municipality Housing and Urban Construction Committee (No. Chengkezi 2019-1-4-2), Forestry Extension Project of China Central Finance (No. Yulinketui 2020-2), the Science Foundation of College of Life Sciences of Southwest University (No. 20212005406201), Ningxia Key Research and Development Project (No. 2020BFG03006), and Ningxia Natural Science Foundation Project (No. 2020AAC03107).

## Conflict of Interest

The authors declare that the research was conducted in the absence of any commercial or financial relationships that could be construed as a potential conflict of interest.

## Publisher's Note

All claims expressed in this article are solely those of the authors and do not necessarily represent those of their affiliated organizations, or those of the publisher, the editors and the reviewers. Any product that may be evaluated in this article, or claim that may be made by its manufacturer, is not guaranteed or endorsed by the publisher.
